# Explaining Continuance Intention of Fruit and Vegetable Consumption among the Rural Elderly: An Application of the Expectancy Confirmation Model

**DOI:** 10.1155/2017/1808475

**Published:** 2017-10-04

**Authors:** Mohamad Reza Jafari, Kambiz Ahmadi Angali, Hashem Mohamadian

**Affiliations:** ^1^Department Health Education and Promotion, Faculty of Health, Ahvaz Jundishapur University of Medical Sciences, Ahvaz, Iran; ^2^Department of Biostatistics and Epidemiology, Faculty of Health, Ahvaz Jundishapur University of Medical Sciences, Ahvaz, Iran; ^3^Research Centre for Social Determinants of Health, Department Health Education and Promotion, Faculty of Health, Ahvaz Jundishapur University of Medical Sciences, Ahvaz, Iran

## Abstract

**Background and Aim:**

Healthy aging is the permanent right of all people. Thus, the purpose of this work was to investigate the expectation confirmation model constructs on intention of continuing to consume fruit and vegetables among the rural elderly.

**Materials and Methods:**

This cross-sectional study was conducted on 332 elderly. The expectation confirmation model served as a theoretical framework. A random sampling was recruited. Data were collected through face-to-face visit in the second 6 months of 2016. The data were then analyzed using LISREL 8.5 and SPSS 16 software.

**Results:**

This model could explain 79% of intention to continue fruit and vegetable consumption. The usefulness and satisfaction had the most effect on the intention to continue the consumption of fruit and vegetables.

**Conclusion:**

Expectation confirmation model depicted a proper application in explaining the intention to continue the consumption of fruit and vegetables in the elderly. Since perceived benefits were the most important factor in determining the intention to continue F&V consumption in this study, it is required that policymakers utilize suitable efficient strategies to promote the perception of benefits of F&V consumption in the elderly by means of campaigns in the society, organizations, and families.

## 1. Introduction 

**Figure 1:**
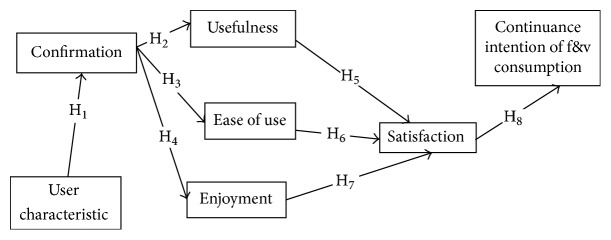
Basic theoretical model of ECM for continuance intention of consuming F&V among the elderly.

World's population is aging [1]. The number of people over the age of 60 is expected to double by 2050 [2]. Therefore, health care for the elderly is one of the most significant health priorities in any country. Since the prevalence of many chronic health conditions increases with age, we might assume that as the population ages there will be progress in the proportion with one or more such conditions, and that their treatment will make expanding demands on the health care system [3]. Chronic diseases are creating a major public health threat worldwide [4]. Chronic disease can engender a loss of independence, years of disability, or death and dictate an abundant economic burden on health services [5]. Low F&V intake is among the top 10 risk factors contributing to global mortality [6]. Worldwide, low intake of F&V is predicted to cause about 19% of gastrointestinal cancer, about 31% of ischemic heart disease, and 11% of stroke. The estimated levels of current F&V intake differ appreciably everywhere in the world ranging from less than 100 g/day in less developed countries to about 450 g/day in Western Europe [7]. Fruit and vegetables (F&V) as part of the daily diet could help prevent major noncommunicable diseases. More than 2.7 million lives could potentially be saved each year with sufficient global F&V consumption [8]. F&V consumption has already been identified as a modification action for chronic diseases prevention and control. WHO recommends intake of a minimum of 400 grams or up to five servings per day F&V as part of a healthy eating pattern for the prevention of chronic diseases [7]. To achieve the WHO recommendation, people should at least double their current intake of F&V. In spite of their numerous health benefits, literature review showed that 77.6% of men and 78.4% of women from fifty-two mainly low- and middle-income countries consumed less than the minimum recommended five daily servings of F&V [9]. Also, there is evidence that older people do not consume enough F&V [10]. Such observations from Iran and elsewhere indicate that there is an urgent need for health education programs in order to increase F&V consumption among the elderly population. Mc Morrow et al. (2016) have emphasized the psychological effects such as habits, beliefs, and lack of knowledge on consumption of F&V [11]. Other research suggests that availability and access to F&V supplies for people living in rural areas is much more limited [12]. To increase F&V consumption the most important components must be identified using a validated theoretical framework [13]. One of the theoretical frameworks affecting acceptance and the continuity of the user's behavior is an Expectancy Confirmation Model (ECM). ECM is widely used in the consumer behavior literature to study consumer satisfaction, postpurchase behavior, and service marketing in general [14]. Bhattacherjee and Premkumar [19] built some modifications on ECM and proposed a model which was, in fact, the modified form of Oliver Model [15]. It is adopted from expectancy value theory. This framework increases the likelihood of implementing more effective interventions and of better understanding the mechanisms responsible for the user's behavior change. Designers and planners of health education are aware of the fact that behaviors will be used continuously by learners whose utility and ease utility have been appreciated by learners. Thus, the ECM would provide us with a proper framework to explain the reasons of F&V consumption in the elderly. Bhattacherjee believes that preutility expectation is seen in satisfaction and appreciation constructs. Also, received function has been removed because its effect is expressed by confirmation construct. In fact, this model considers the user continuance intention in the three subscales of perceived benefits, expectancy confirmation, and users' satisfaction. Recently, constructs of ease of use and perceived enjoyment have been added to the model. Furthermore, effect of the individual characteristics as a dimension added to the ECM was taken into consideration in the analysis. All these variables were tested as predictors and as moderators of the intention of continuing F&V consumption. To date, no study has investigated F&V continuance intention consumption within this specific theoretical framework. Explaining important factors affecting F&V consumption among the Iranian rural elderly is a necessary step in the development of an effective educational intervention. Therefore, aim of the study was to test the ECM model predictors for continuance intention of F&V consumption among a sample of the Iranian rural elderly and to explore the potential of background factors (user characteristics) to test the direct and moderating effects of the variables within the ECM framework, as displayed in Figure 1. Therefore, consistent with the theory and based on the previous findings, we suggest that

(H1) user's demographic characteristics would significantly affect his/her perceived confirmation to continuance intention of consuming F&V;
(H2) perceived confirmation would significantly affect the usefulness to continuance intention of consuming F&V;
(H3) perceived confirmation would significantly affect the ease of use of continuance intention of consuming F&V;
(H4) perceived confirmation would significantly affect the perceived enjoyment of continuance intention of consuming F&V;
(H5) usefulness would significantly affect satisfaction regarding continuance intention of consuming F&V;
(H6) ease of use would significantly affect satisfaction regarding continuous intention of consuming F&V;
(H7) perceived enjoyment would significantly affect satisfaction regarding continuance intention of consuming F&V;
(H8) satisfaction would significantly predict continuance intention of consuming F&V.

## 2. Methods

### 2.1. Data Collection and Sample

This cross-sectional study included 332 elderly from health centers in Abadan, Iran. The data were gathered during September 2015 and March with face-to-face interviews implemented by four trained and experienced interviewers. Total population of 60-year-olds and over was 10598I which included 5392 males and 5206 females. 3066 out of this were the rural inhabitants including 1388 males and 1678 females. Participation was free of cost and all the individuals aged 60 and above were fit to join.

A random sample was recruited from all of the listed people. The sample size was calculated on the basis of cross-sectional studies proportion design [16]. Salehi et al. have guessed that at best 50% of the elderly would have an inadequate FV intake [10]. A sample of 332 elderly attained for this study, which was more than 200 samples, was recommended in Structural Equation Modeling (SEM) literatures [17]. Those who did not agree to pursue the study and those who were suffering from serious illness up to 1 month before the date of data gathering were excluded.

The ethics board of the Ahvaz Jundishapur University of Medical Sciences approved the study with the number of SDH-9509. Before the study was done, the aim, method, and confidentiality were disclosed fully to the participants and if they were gratified to take part they were asked to read and sign a consent form. The principals of all the health centers were also approached about their willingness to participate.

#### 2.1.1. Measures

In the present study we used several instruments to collect the data.

#### 2.1.2. Demographic Questionnaire

1. This comprised information on age, gender, F&V eating experience, financial independence, history of disease, physical activity, smoking, self-efficacy, and so on.
2. Six constructs of the ECM were measured in this study: continuance intention of consuming F&V, satisfaction, usefulness, ease of use, perceived enjoyment, and perceived confirmation, respectively. The constructs were measured using multiple-item scales.

We adapted scales demonstrating good psychometric properties in previous studies. The 4-item scale for the continuance intention of F&V consumption was adapted from Hsu et al. (2004) [18] and Bhattacherjee and Premkumar (2004) [19], and the 2-item scale for satisfaction was adapted from Spreng and Page Jr. (2003) [20] and Bhattacherjee/Premkumar (2004) [19]. The 4-item scale for ease of use was adapted from Davis (1993) [21]. The 6-item scale for usefulness was adapted from Davis (1993) [21]. The 5-item scale for perceived enjoyment was adapted from Davis (1993) [21], and the 6-item scale for perceived confirmation was adapted from the Bhattacherjee [14].

In order to define the scale content validity in the study, both qualitative and quantitative methods were used. In qualitative content validity, the questionnaire was given to a group of health education experts with three years of work experience and/or with scientific paper publication. Qualitative content validity included compliance with grammar, use of proper vocabulary, importance of items, and proper arrangement of items in their own places. After gathering the experts' opinions, the required modifications were applied on tools by consultation with members of the research team. In quantitative content validity, the opinions of 8 relevant experts were used. In order to make sure of selecting the most important and correct content (necessity of item), content validity ratio (CVR) was used. Also, content validity index (CVI) was used to make sure that tool items were designed in the most suitable way to measure it [22]. Experienced experts of content validity were asked to answer the tool items using one of the following options: (a) necessary, (b) useful but not necessary, and (c) not necessary. The answers were calculated according to CVR equation; if item scores were higher than the number of Lawshe's table (0.75) [23], it indicated that the presence of relevant items in this tool, with an acceptable level of statistical significance, would be essential. Then, after determining CVR, the questionnaire was again given to the expert team for CVI determination. They were asked to express their views about each item according to a four-point Likert scale. Then, using CVI equation, the CVI was calculated.

An SEM technique was used on the data that were collected to test for the model identified in Figure 1. SEM can be theoretically employed to respond to every research inquiry. SEM is a statistical approach for concurrently inquiring about and predicting a set of hypothesized relationships among numerous independent and dependent variables [24]. The coefficient of determination *R*-square was used to measure the explained variance of the endogenous variables. Endogenous variables are affected by other variables. But, exogenous variables are not explained within the model. SEM assists the investigator in replying to a set of interrelated research concerns in a single, systematic, and comprehensive analysis. SEM can demonstrate to us if our model is adequate or not. One of the most valuable processes in SEM is appraising whether a specified model “fits” the collected data or not [25]. Sum of goodness-of-fit indices have been established to evaluate SEM models. Model fit was assessed with chi-square (*χ*^2^), comparative fix index (CFI), goodness-of-fit index (GFI), the Root mean square error of approximation (RMSEA), normed fit index (NFI), and the standardized root mean square residual (SRMR) [26]. A structural model (path diagram) is a part of the entire SEM diagram. It is used to relate all of the variables (both exogenous and endogenous) to account for in the model. Using Lisrel 8.5 [27], it is possible to examine the influence of many variables on other variables according to a specified model. Thus, the whole ECM can be tested in relation to the dataset in one analysis.

Before analysis, all variables were evaluated to confirm the accuracy of input data and multivariable analysis assumptions.

## 3. Results

The study population consisted of 54.4% males and 45.6% females with the age range of 60–87 years and mean age of 67.45 ± 6.1 years. The majority (53.1%) were illiterate. Almost half of them (47%) had an income of less than 50,000 USD a month. 48.1% of them ate 1–3 servings of F&V per day. On the contrary, almost 36% of them had no F&V intake per day. Nonsmokers were 70.3% of the participants. Finally, 55.6% cases had no physical activity. Characteristics of the population are shown in Table 1.

Since multicollinearity is a problem with the criteria or tolerance > .20 or variance inflation factor (VIF) < 5 [28], in this study, there was no inconsistency for SEM because the evaluation of multicollinearity test showed that the tolerance of predicting variables and VIF had fluctuated between 0.810 and 1.00 and 1.00 and 1.234, respectively.

We calculated the means and standard deviations for each variable and created a correlation matrix as shown in Table 2.

The final model showed a good model fit (*ϰ*^2^ = 57.09, *P* = 0.51, CFI = 1.00, GFI = 0.97, RMSEA = 0.001). Additionally, a summary of indices and the recommended values are given in Table 3.

**Figure 2:**
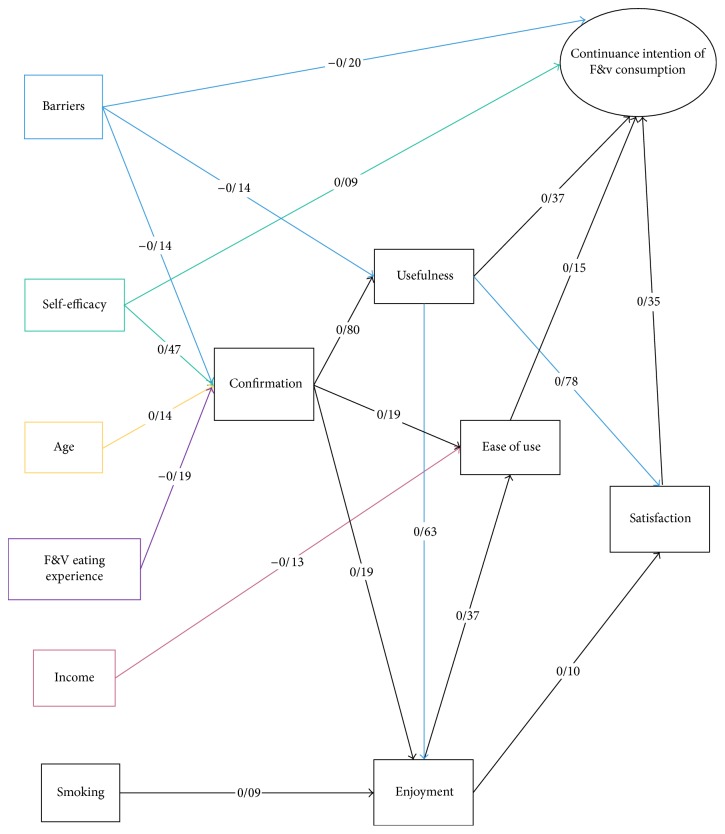
Final model of ECM for continuance intention of consuming F&V among the elderly.

The test results of the path analysis are summarized in Figure 2 showing that user's demographic characteristics (age, F&V eating experience, income, self-efficacy, and perceived barriers) had a positive direct influence on confirmation, which explains 28% of the confirmation variance. Also, perceived ease of use is predicted by confirmation, income, and perceived enjoyment directly and self-efficacy, perceived barriers, age, F&V eating experience, perceived usefulness, and smoking indirectly, which explains 30% of its variance. As expected, perceived enjoyment can be predicted by confirmation, perceived usefulness, and smoking directly and age, self-efficacy, F&V eating experience, perceived barriers, and confirmation indirectly which explains 64% of its variance. Additionally, perceived usefulness is predicted by perceived barriers and confirmation directly and age, self-efficacy, perceived barriers, and F&V eating experience indirectly which explains 71% of its variance. Also, satisfaction is predicted by perceived usefulness and perceived enjoyment directly and age, self-efficacy, perceived barriers, smoking, F&V eating experience, and confirmation indirectly which explains 73% of its variance. Finally, the continuance intention of consuming F&V is predicted by perceived barriers, self-efficacy, ease of use, perceived usefulness, and satisfaction directly and perceived barriers, self-efficacy, age, income, F&V eating experience, smoking, confirmation, perceived enjoyment, and perceived usefulness indirectly which explains 79% of its variance.

In summary, all path coefficients in the final model were significant with the exception of the path from ease of use to satisfaction (H6).

## 4. Discussion

When exploring continuance intention to consume F&V based on ECM theoretical framework in the current study, the findings revealed that (1) the most influential determinant of confirmation is self-efficacy, (2) the most influential reason of ease of use is confirmation, (3) the most influential explanation of perceived enjoyment is confirmation, (4) the most influential factor of usefulness is confirmation, (5) the most influential determinant of satisfaction is usefulness, and, lastly, (6) the most noticeable reason of continuance intention of consuming F&V is usefulness.

The findings approve the appropriateness of the ECM in explaining the intention of continuing consumption of F&V among the elderly; the ECM variables along with demographic factors were able to forecast 79% of intention of continuing consumption F&V. This issue is accordant with Lee's (2010) study [29]. Additionally, the rate of explaining of variance acquired from this study was more than that in Guillaumie et al.'s study [30]. Also, Babak et al. (2014) reported that the intention of F&V consumption using the theory of planned behavior framework was 35% [31].

Although cross-sectional studies are acknowledged to blow up the relationship between psychosocial variables and behavior, a meta-analysis of F&V consumption reasons has demonstrated a greater adequacy in explanation during utilizing a long-term study. But, the researchers suggested that their multiple results provide fair support for the greater efficacy in predicting when good psychometric quality tools were utilized to evaluate psychosocial and behavioral scales.

Intention is the principal determinant in discerning continuing behavior, indicating that F&V consumption among the elderly is robustly changed by rational debate. Other findings ratify why the intention is an important reason in F&V consumption.

This study has shown that background characteristics affect intentions by mediation of confirmation. Hence, it is recommend to adjust these factors during doing subsequent studies.

As stated in the ECM, interventions must be conducted at alterable crucial beliefs in order to offer visible changes in confirmation, ease of use, perceived enjoyment, perceived usefulness, and satisfaction so able to create further influence on intentions in the wanted direction.

Nevertheless, the intervention would be efficient if people constitute their goals based on rational intentions. It would occur only if there was a substantial connection between intention and behavior. Thus, while it is proper to target an intervention at any one of the main constructs in the ECM, it may be prudent to regard their relative weights in the explanation of intention and behavior to target the intervention.

The results of our study were consistent with Bhattacherjee's study [14], showing that perceived usefulness had notable influences upon continuance intention of consuming F&V. Also, the findings of this study were consistent with Mirheidari et al. [32]. Huang and Chang showed that the most convincing factor in impacting consumers' intention to shop on foreign online stores was the perceived benefits [33], but the results of Cheng [34], Stone and Baker-Eveleth [35], and Lee's [29] studies showed that user's satisfaction had a greater effect on the intention of continuing consumption, which was in disagreement with the findings of the current study.

To ameliorate the perceived usefulness of consuming F&V it would be likely to yield mandates or “tips” on buying, depository, and readiness of diverse varieties of F&V involving information about places to get low-cost F&V and prompt and simple recipes. Giving accurate information on the material consequences of consuming F&V routinely, especially short-term health consequences, may better affect elderly's continuance intention of eating F&V.

The perceived usefulness is referred to as individual perception of the acquired advantages of F&V consumption. Perceived usefulness in the elderly would be appreciated by comparing the tangible benefits of F&V consumption with previous perceptions of its usefulness. If the elderly find out that F&V consumption would be as much as he/she had thought, the satisfaction could be achieved. The elderly satisfied with F&V intake will intend to continue consumption, while those unsatisfied will stop it.

If they understand the usefulness of F&V consumption, there would be no need to confirm it; instead, without confirming, they would instantly accept the intention of F&V consumption. Thus, perception of F&V consumption usefulness will lead to continuous consumption. Furthermore, this confirmation would have a long-term effect on the perception of benefits after experiencing the F&V consumption.

After a period of F&V intake, the individuals' perception of the benefits of F&V intake would be based on the primary expectation and, thus, the continuation of F&V consumption depends on meeting their expectations. If its benefits attain a higher level than their expectations, they will continue F&V consumption. But, if it attains a lower level than their expectation, the intention of continuous consumption will be stopped.

In this study, self-efficacy had the lowest level of explanation among variables with a direct relationship to the intention of continuous F&V consumption, but, in other studies [36–38], it was reported as the most important explanation of behavioral intention. Self-efficacy relies on the individual skills of perception and abilities in successful performance of intended functions. Perhaps the higher the age of the elderly, the higher the risk of physiocognitive disorders and the lower the level of physical functions and the higher the level of dependence in daily activities, which affects their self-efficacy. Thong et al. [39] showed that perceived enjoyment and ease of use had considerably affected the user's intention of continuous consumption. It was compatible with this study. Ease of use refers to the individual's perception of usefulness, affected by the fact that he/she must perceive the feasibility of F&V consumption. In fact, those elderly people who are aware of the benefits of F&V consumption for the health and intend to continue consuming these food groups will consume them feasibly. It means that awareness of the benefits of F&V consumption could create more motivation to continue the consumption and, the perception of ease of use in the elderly will persuade them to consume more F&V.

Literature review revealed that financial problems could be regarded as the major hindering of F&V consumption [40]. Owing to financial reasons such as high costs, insufficient income, lack of feasibility, and periodontal problems, the elderly may conceive F&V consumption as costly, difficult, and unpleasant [41]. As a barrier to performing a behavior, the perceived barriers may reduce the practice and intention of F&V consumption in the elderly.

Ultimately, the findings of this study revealed that the expectation of F&V consumption was confirmed in the elderly. It led to the elderly being aware of the effectiveness of benefits of F&V consumption against its barriers and institutionalizing the intention of continuing F&V consumption. The information discovered of this investigation might not be generalized to other Iranian elderly because our participants were rural elderly. These elderly might be distinguishable from others concerning social and economic factors, convenience, and opportunity of F&V consumption. More enquiries are necessitated to scrutinize the conciliating reasons influencing F&V in a culturally diverse population of Iranian rural elderly. Moreover, it is necessary to note that our results on F&V intake were formed on self-reported data and consequently could be related to appraisal mistakes. Many features were not explored in this study; because season could impact the F&V consumption, it is proposed that it should be contemplated in future investigations.

## 5. Conclusion

The perceived benefits were the most important factors in determining the intention of continuous F&V consumption in this study; it is required that policymakers utilize suitable efficient strategies to promote the perception of benefits of F&V consumption in the elderly by means of campaigns in the society, organizations, and families and, thereby, increasingly sensitize the individuals regarding the aforementioned benefits. Therefore, broadcasting audiovisual educational TV/radio programs can be effective. Additionally, setting up accessible, organic F&V marketplaces will predispose the intention of continuous F&V consumption. Ultimately, the methodology of this study can be considered in other populations as an educational intervention approach.

## Figures and Tables

**Table 1:** Characteristics of respondents.

Variable	Category	*N*	%
Age, y	60–70	212	66/2
	70–80	90	28/2
	>80	18	5/6

Educational level	Illiteracy	170	53/1
	Primary	109	34/1
	Secondary	28	8/8
	Diploma or higher	13	4

Sex	Male	174	54/4
	Female	146	45/6

Income	Good	17	5/3
	No Good & No Bad	170	53/1
	Bad	133	41/6

F&V eating experience	1–3 servings per day	154	48/1
	3–5 servings per day	45	14/1
	>5 servings per day	6	1/9
	None	115	35/9

Smoking	Yes	95	29/7
	No	225	70/3

Physical activity	<15 minutes	71	22/2
	15–30 minutes	46	14/4
	>30 minutes	25	7/8
	None	178	55/6

**Table 2:** Pearson correlation matrix among continuous variables.

Variables	(1)	(2)	(3)	(4)	(5)	(6)	(7)	(8)	(9)	(10)	(11)	(12)
(1) Self-efficacy	1											
(2) Confirmation	0/452^*∗∗*^	1										
(3) Ease of use	0/418^*∗∗*^	0/724^*∗∗*^	1									
(4) Usefulness	0/398^*∗∗*^	0/828^*∗∗*^	0/829^*∗∗*^	1								
(5) Satisfaction	0/381^*∗∗*^	0/706^*∗∗*^	0/666^*∗∗*^	0/780^*∗∗*^	1							
(6) Enjoyment	0/336^*∗∗*^	0/745^*∗∗*^	0/814^*∗∗*^	0/853^*∗∗*^	0/704^*∗∗*^	1						
(7) Barriers	−0/91	−0/18^*∗∗*^	−0/428^*∗∗*^	−0/289^*∗∗*^	−0/239^*∗∗*^	−0/266^*∗∗*^	1					
(8) Age	−0/117^*∗*^	0/73	0/77	0/001	−0/002	−0/005	0/72	1				
(9) Income	−0/017	−0/060	−0/067	−0/058	−0/35	−0/097	0/020	0/03	1			
(10) F&V eating experience	0/054	−0/017^*∗∗*^	−0/196^*∗∗*^	−0/172^*∗∗*^	−0/107	−0/222^*∗∗*^	0/050	−0/008	0/245^*∗∗*^	1		
(11) Smoking	0/017	0/032	0/056	0/051	−0/047	0/022	0/024	0/038	−0/004	−0/253^*∗∗*^	1	
(12) Intention	0/290^*∗∗*^	0/461^*∗∗*^	0/511^*∗∗*^	0/437^*∗∗*^	0/508^*∗∗*^	0/424^*∗∗*^	−0/120^*∗*^	0/071	0/159^*∗∗*^	−0/038	−0/069	1
Mean	22/67	18/98	12/54	19/75	6/54	15/87	18/06	67/45	—	—	—	13/16
SD	5/82	5/88	4/73	7/35	1/44	6/28	4/69	6/1	—	—	—	3/50
Range	7–35	6–30	4–20	6–30	2–10	5–25	6–29	60–87	—	—	—	4–20
*α*	0/78	0/92	0/84	0/95	0/88	0/91	0/68	—	—	—	—	0/82

^*∗*^ *P* < 0.05, ^*∗∗*^*P* < 0.01.

**Table 3:** Indices and the recommended values of model fit for the structural model.

Indices of model fit	Recommended value	The final model	Conclusion
*P* value	*P* ≥ 0/05	*P* = 0/51	Fit
Chi-squared test/d.f.	<3.00	0/984	Fit
Goodness-of-fit	≥0/90	0/97	Fit
Adjusted goodness-of-fit	≥0/80	0/96	Fit
Normed Fit Index	≥0/90	0/98	Fit
Comparative Fit Index	≥0/90	1/00	Fit
Root mean square residual	≤0/09	0/30	Fit
Root mean square error of approximation	≤0/10	0/0001	Fit
